# Palmitoleic (16:1 n-7) acid and metabolic health: integrating observational, clinical, and mechanistic evidence

**DOI:** 10.3389/fnut.2026.1801946

**Published:** 2026-05-20

**Authors:** Frédéric Destaillats, Manuel Oliveira, Xiaoying Zhou, Mona Correa, James Casey Lippmeier, Leon Parker, Walter Rakistky

**Affiliations:** Checkerspot, Inc., Alameda, CA, United States

**Keywords:** cardiometabolic risk, *de novo* lipogenesis, dyslipidemia, inflammation, insulin resistance, lipokine, non-alcoholic fatty liver disease, palmitoleic acid

## Abstract

Palmitoleic acid (POA; 16:1 n-7) has been proposed as a lipokine linking adipose tissue, liver, and skeletal muscle metabolism, but its relevance to human metabolic health remains uncertain. This narrative review integrates observational, clinical, and mechanistic evidence across circulating lipids and lipoproteins, glucose homeostasis, inflammation and vascular markers, adiposity, and hepatic endpoints. In observational studies, higher POA in esterified lipid fractions often tracks with *de novo* lipogenesis, hypertriglyceridemia, adiposity, insulin resistance, and fatty liver, whereas findings for free POA are more heterogeneous. In human interventions, POA-rich food matrices such as macadamia-based diets can improve LDL-cholesterol and related ratios when they replace saturated fat, but these studies do not isolate the specific contribution of POA from the broader monounsaturated-fat matrix. By contrast, short-term trials of purified POA supplementation have largely reported neutral effects on standard fasting lipids, glycemic markers, and inflammatory biomarkers. Pre-clinical and *in vitro* studies support biological plausibility, with reported effects on insulin signaling, hepatic lipid metabolism, adipose remodeling, and inflammatory pathways. Overall, current evidence supports cautious interpretation: POA is biologically interesting, but clinically meaningful benefits of purified POA supplementation remain unproven and require adequately powered trials in well-characterized at-risk populations.

## 1 Introduction

Palmitoleic acid (POA; 16:1 n-7 or *cis*-9 16:1) is a monounsaturated fatty acid produced primarily through stearoyl-CoA desaturase-1 (SCD1)-mediated desaturation of palmitic acid. It has been proposed as a lipid mediator, or lipokine, linking adipose tissue metabolism with insulin sensitivity and hepatic lipid handling (1, 2). A key interpretive issue is that circulating POA can arise both endogenously, as part of SCD1-driven lipogenesis, and exogenously, from diet or supplements. These sources do not necessarily reflect the same biology. This distinction helps explain why higher POA in cholesterol esters, phospholipids, or adipose tissue is often associated with hypertriglyceridemia, abdominal adiposity, insulin resistance, non-alcoholic fatty liver disease, and vascular risk, whereas some studies of free POA or specific phospholipid pools report associations with better insulin sensitivity or lower diabetes risk (3–10).

Only a few foods provide significant amounts of POA. Seabuckthorn (*Hippophae rhamnoides*) berry pulp and peel oil is the richest botanical source, whereas sea buckthorn seed oil contains much less POA and is richer in linoleic and α-linolenic acids (11–15). Macadamia oil is another major plant source, and avocado oil provides intermediate amounts (2, 15–18). Salmon and cod liver oil provide ≈6–7% POA or fish-oil–derived concentrates such as 7-MEGA™ or Provinal™ reach ~50% POA as ethyl esters. Smaller amounts occur in ruminant fat, where POA is present mainly as *trans*-9 16:1 and often serves as a biomarker of dairy fat intake rather than a major contributor to total fatty acid exposure (2, 19–25). Most conventional vegetable oils contribute little POA (2, 15). More recently, Zhou et al. (26) have proposed controlled microalgal fermentation to produce an oil with >50% POA, offering a year-round, land-efficient, vegetarian, and compositionally consistent source of high-purity POA.

The sections below organize the evidence by metabolic domain and emphasize the need to distinguish endogenous POA as a marker of lipogenesis from exogenous POA delivered in foods or supplements.

## 2 Literature search strategy

This manuscript is a narrative review. Relevant studies were identified primarily through PubMed and Google Scholar through March 2026 using combinations of the terms “palmitoleic acid”, “palmitoleate”, “16:1 n7”, “omega7”, “macadamia”, “seabuckthorn”, “lipokine”, “lipids”, “glucose”, “insulin resistance”, “inflammation”, “NAFLD”, “blood pressure”, and “adiposity”. Reference lists of relevant reviews and original articles were also screened to identify additional human observational studies, intervention trials, and mechanistic pre-clinical reports. Priority was given to peer-reviewed human studies addressing cardiometabolic outcomes, while animal and cell studies were included to provide mechanistic context. Because this is a narrative rather than systematic review, studies were not formally graded, but retracted reports and major design limitations were considered explicitly in the interpretation of the evidence.

## 3 Effects on circulating lipids and lipoproteins

### 3.1 Observational and cohort studies

Observational data in diverse populations indicate that higher endogenous POA generally correlates with an atherogenic lipid profile and with indices of hepatic *de novo* lipogenesis. In the Cardiovascular Health Study, a prospective cohort of 3630 older US adults, higher plasma phospholipid POA was independently associated with lower LDL-cholesterol and lower total: HDL-cholesterol ratio, but also with higher triglycerides (TG) and, in men, greater insulin resistance; despite these mixed associations with risk factors, POA was not significantly related to the incidence of type-2 diabetes over a median 13 years of follow-up (4). In 134 healthy French men, plasma POA content, used as a surrogate of SCD1 activity, was correlated with fasting TG (r~0.53), waist circumference, and systolic blood pressure; men in the highest vs. lowest quartile of plasma POA had ~2-fold higher triglyceridemia, and POA remained independently associated with both hypertriglyceridemia and abdominal adiposity in multivariable models (3).

In a large cross-sectional study of middle-aged and older Chinese adults, erythrocyte POA was positively associated with TG, total cholesterol, blood pressure, fasting insulin, HOMA-IR, several pro-atherogenic adipokines (RBP-4, PAI-1) and hs-CRP, and inversely associated with HDL-cholesterol and adiponectin; mean erythrocyte POA increased stepwise with the number of metabolic syndrome components, supporting a close link between higher POA and an adverse cardiometabolic profile (5). Consistent with POA reflecting increased lipogenesis, lipidomic profiling of patients with non-alcoholic fatty liver disease showed that both simple steatosis and NASH were characterized by higher circulating POA and related 16:1 acid isomers across several lipid classes compared with lean controls, consistent with upregulated SCD1 activity and *de novo* lipogenesis in NAFLD (8). Finally, case-control analyses in US male physicians reported that higher red-blood-cell POA was associated with increased risks of coronary heart disease and incident heart failure over more than two decades of follow-up, even after adjustment for traditional risk factors, suggesting that chronically elevated POA may reflect long-term vascular risk (7).

These observational findings suggest that higher circulating POA, particularly in esterified lipid fractions, is more consistent with an SCD1-driven lipogenic state than with a uniformly beneficial metabolic signal, providing a rationale for controlled dietary intervention studies.

### 3.2 Dietary interventions

Curb et al. (18) conducted a randomized, three-period crossover trial in 30 generally healthy men and women aged 18–53 years in Hawaii, USA (Table 1). Participants consumed for 30 days each: a “typical American” high-saturated-fat diet (37% energy from fat), an American Heart Association Step 1 diet (30% energy from fat, lower in saturated fatty acids), and a macadamia-nut-based diet providing 37% energy from fat, mostly monounsaturated fatty acids (MUFA), including both oleic acid and POA from macadamia nuts (18). Relative to the typical American diet, both the Step 1 diet and the macadamia-nut diet significantly lowered total and LDL-cholesterol, despite the macadamia diet having the same total fat content as the typical diet (18). HDL-cholesterol fell slightly on the Step 1 and macadamia diets compared with the typical diet, but TG concentrations were not adversely affected, and overall, the macadamia diet produced a lipid profile comparable to the Step 1 low-fat diet (18). These data suggest that substituting saturated fat for macadamia-derived MUFA, including POA, improves LDL-cholesterol even when total fat remains relatively high (18).

**Table 1:** Selected human intervention studies evaluating dietary palmitoleic acid (POA; 16:1 n-7) intake from foods or supplements and associated cardiometabolic or inflammatory outcomes.

Ref.	Country	Population	Source of dietary POA	Daily dose of POA	Measured outcomes	Main findings
(18)	USA	30 healthy adults (18–53 y)	Macadamia-nut-enriched foods	~2,000–3,000 mg/day POA	Serum lipids	Lower total and LDL-C similarly to a Step-1 low-fat diet; slight reduction in HDL-C; TG unchanged.
(28)	Australia	17 hypercholesterolemic men	Macadamia nuts	~540–1,230 mg/day POA	Serum lipids, body weight, plasma fatty acids	Lower total-C and LDL-C; higher HDL-C; TG unchanged; slight weight reduction; plasma POA increased.
(27)	USA	25 mildly hypercholesterolemic adults	Macadamia nuts	~580 mg/day POA	Lipid/lipoprotein profile	Lower total-C, LDL-C, non-HDL-C, and lipid ratios; TG unchanged; weight maintained.
(29)	Japan	71 healthy young women	Macadamia-nut-enriched bread	~570 mg/day POA	Serum lipids, body weight	Lower total-C and LDL-C; lower body weight; erythrocyte POA increased; TG and HDL-C unchanged.
(30)	USA	35 adults with abdominal obesity	Macadamia nuts (~15% of energy)	Individualized (~15% of energy)	Body weight, body composition, lipids, glycemic parameters	No significant changes in weight, body fat, glycemic parameters, or lipids overall; non-significant reductions in total-C and LDL-C, greater among participants with lower adiposity.
(31)	Australia	34 hypercholesterolemic men	Macadamia oil-rich margarines compared with palm and high-oleic oils	~1,050 mg/day POA	Serum lipids	LDL-C on the POA diet was similar to the palmitic-acid diet and higher than the oleic-acid diet.
(32)	India	74 hypertensive/hypercholesterolemic men plus 32 normotensive controls	Sea buckthorn seed oil (not pulp oil)	~35 mg/day POA	Blood pressure, lipids, oxidized LDL, antioxidant capacity	Lower BP, total-C, LDL-C, TG, and oxidized LDL in hypertensive subjects; antioxidant capacity increased. POA-specific contribution cannot be isolated.
(33)	USA	13 overweight/obese adults	Sea buckthorn berry-oil-derived capsules (*cis*-POA or *trans*-POA-enriched)	POA: 380–1,520 mg/day; *trans*-POA: 120–480 mg/day	PLFA levels, glucose, lipids, clinical measures	Dose-dependent increase in circulating POA isomers; no significant changes in glucose, insulin, or lipids.
(34)	USA	123 adults with hs-CRP >=2 mg/L	Fish-oil-based POA supplement	500 or 1,000 mg/day	hs-CRP, IL-6, TNF-α, glucose, insulin, lipids, satiety hormones	No significant effect on hs-CRP, cytokines, lipids, glucose, insulin, or satiety hormones; plasma POA increased.
(35)	USA	40 overweight/obese adults with prediabetes	High-purity POA capsules (>90% POA as ethyl esters)	1,512 mg/day	Planned: insulin sensitivity (clamp), liver fat (MRI), adiposity, lipids, cytokines	Ongoing trial; no results available.
(49)	USA	50 adults with chronic musculoskeletal discomfort	Mixed omega-7 fatty acid supplement	688 mg/day palmitoleate	hs-CRP, IL-6, TNF-α, self-reported outcomes	No reduction in inflammatory biomarkers over 3 weeks compared with placebo.

The published sea buckthorn trials evaluated different oil fractions; Vashishtha et al. (32) studied seed oil, whereas Huang et al. (33) used a berry-oil-derived preparation.

BP, blood pressure; C, cholesterol; HDL, high-density lipoprotein; hs-CRP, high-sensitivity C-reactive protein; IL-6, interleukin-6; LDL, low-density lipoprotein; MRI, magnetic resonance imaging; PLFA, phospholipid fatty acid(s); POA, palmitoleic acid; TG, triglycerides; TNF-α, tumor necrosis factor α.

Garg et al. (27) studied 17 middle-aged hypercholesterolemic men in Australia in a single-arm, 4-week intervention trial (Table 1). Participants consumed freshly roasted macadamia nuts providing 40–90 g/day, corresponding to ~15% of total energy, without other prescribed dietary changes (28). After 4 weeks, total cholesterol was lower by about 3%, LDL-cholesterol by ~5%, and HDL-cholesterol increased by nearly 8%, with no significant change in TG or homocysteine 30. Levels of plasma POA phospholipid and other MUFA increased, confirming the dietary intake of POA-rich fat (28). Body weight and BMI showed very small but statistically significant reductions, suggesting that macadamia nuts did not promote weight gain in this context (28).

Griel et al. (27) performed a controlled, randomized cross-over feeding study in 25 mildly hypercholesterolemic men and women in USA (Table 1). Participants consumed, in random order, two 5-week isoenergetic diets: an “average American diet” (AAD; 33% energy from fat, 13% saturated, 11% MUFA) and a macadamia-nut-rich diet (MAC; 33% fat, 7% saturated, 18% MUFA) that supplied 42.5 g/day macadamia nuts, providing ~56% of energy from oleic acid and ~14% from POA (27). Compared with the AAD, the MAC diet significantly reduced total cholesterol and LDL-cholesterol (approximately 9–10% lower) and also lowered non-HDL-cholesterol and the total-/HDL-cholesterol and LDL-/HDL-cholesterol ratios, while TG remained unchanged (27). Body weight was maintained by design, showing that cardioprotective lipid changes occurred independently of weight loss (27).

In Japan, Hiraoka-Yamamoto et al. (29) randomized 71 healthy young women (19–23 years) to consume, for 3 weeks, breads enriched with either macadamia nuts, coconut or butter (Table 1). Each bread provided 10 g of test fat per slice, and participants consumed two slices per day; the macadamia bread contained 70.5% of its fat as MUFA and ~2.85 g POA per 100 g bread, leading to a modest daily POA dose when two loaves were consumed (29). After 3 weeks, total and LDL-cholesterol fell significantly from baseline in both the macadamia and coconut groups, whereas no significant change occurred in the butter group (29). HDL-cholesterol, TG and free fatty acids were unchanged in all groups, while body weight and BMI decreased significantly only in the macadamia group, and blood pressure remained stable (29). Erythrocyte POA increased significantly with macadamia bread, indicating incorporation of dietary POA into circulating lipids (29).

Taken together, these macadamia studies suggest that replacing saturated fat with a macadamia-based monounsaturated-fat pattern can lower LDL-cholesterol in some settings. However, these interventions do not isolate POA from oleic acid or other features of the nut matrix. Consistent with this more cautious interpretation, a later 8-week randomized crossover trial in 35 adults with abdominal obesity found no significant changes in weight, body fat, glycemic parameters, or lipids overall, although non-significant reductions in total and LDL-cholesterol were observed and appeared greater in participants with lower adiposity (30).

Nestel et al. (31) examined the effects of increasing dietary POA using macadamia oil compared with palm oil (palmitic acid) and high-oleic sunflower oil in hypercholesterolemic men in Australia (Table 1). In a four-period crossover design (one baseline, three 3-week test periods), participants consumed prepared drinks and margarines containing macadamia oil (POA-rich), palm oil (palmitic acid rich) or high-oleic oil (oleic acid rich), with the supplements contributing about 17% of energy as POA plus palmitic acid on the POA diet, similar to the oleic contribution on the oleic diet (31). The oleic-acid diet produced significantly lower total and LDL-cholesterol than either the palmitic or POA diets (31). LDL-cholesterol on the POA diet was very similar to that on the palmitic acid diet, indicating that macadamia oil, when used as a major dietary fat, behaves more like palmitic than like oleic acid with respect to LDL-cholesterol (31). HDL-cholesterol tended to be lower with the POA diet than with the palmitic diet (31).

Vashishtha et al. (32) studied seabuckthorn seed oil, not the more POA-rich pulp oil. The seed oil contains only about 4.9% POA and also substantial linoleic, α-linolenic, and oleic acids, plus vitamin E and carotenoids, making it a mixed intervention rather than a high-POA botanical oil (Table 1). In this randomized, double-blind trial, 32 normotensive normocholesterolemic men (cohort 1) and 74 hypertensive, hypercholesterolemic men (cohorts 2 and 3) were recruited; hypertensive groups were assigned to seabuckthorn seed oil capsules (0.75 mL/day) or sunflower-oil placebo for 30 days, while the normotensive group received seabuckthorn seed oil capsules only (32). In hypertensive hypercholesterolemic subjects, seabuckthorn supplementation led to significant reductions in systolic and diastolic blood pressure and marked decreases in total cholesterol, LDL-cholesterol, TG and oxidized LDL compared with baseline and relative to placebo, whereas effects in normotensive normocholesterolemic subjects were smaller (32). Circulating total antioxidant capacity increased in both normal and hypertensive subjects, consistent with the combined effects of unsaturated fatty acids and antioxidant micronutrients in seabuckthorn oil (32). Because POA is a component of this oil and the product also contains linoleic acid, α-linolenic acid and antioxidant compounds, the specific contribution of POA to the lipid-lowering effect cannot be isolated (32).

In contrast to the seed-oil trial above, Huang et al. (33) performed a randomized, double-blind, crossover dose-escalation trial in 13 metabolically healthy but overweight or obese adults in the United States using unmodified seabuckthorn berry oil relatively rich in *cis*-POA and an isomerized preparation augmented in *trans*-POA (Table 1). Participants received escalating doses of either unmodified seabuckthorn oil rich in POA (380, 760, and 1,520 mg/day) or isomerized seabuckthorn oil augmented in *trans*-POA (120, 240, and 480 mg/day), each for three 3-week periods separated by a 4-week washout (33). The primary outcome, serum phospholipid POA isomers, increased modestly and dose-dependently: *trans*-POA rose by about 15–27% at the highest augmented dose, whereas POA (*cis* isomer) showed only a modest positive trend with the unmodified oil (33). Importantly, despite these clear changes in circulating POA isomers, there were no significant effects on fasting serum cholesterol, TG or other lipid parameters in this small trial, and all values remained within normal ranges (33).

A more recent, larger trial by Bridges et al. (34) randomized 123 adults in North Carolina, USA, all with hs-CRP >=2 mg/L, to 12 weeks of 500 mg/day or 1,000 mg/day marine-derived POA or olive-oil placebo (Table 1). The POA capsules were highly enriched in POA (~50% of capsule content) and virtually free of long-chain n-3 fatty acids, and red blood cell and plasma POA levels confirmed good compliance and dose-dependent increases in circulating POA (34). Despite effectively raising POA status, Bridges et al. (34) did not observe significant changes in LDL-cholesterol, total cholesterol, HDL-cholesterol, or TG relative to placebo, and responses did not differ by baseline hs-CRP levels. These findings indicate that, at doses up to 1 g/day, purified POA does not materially alter fasting lipids in generally healthy but inflamed adults over 12 weeks.

Cetin et al. (35) report the published protocol of a U.S.-based clinical trial that is currently ongoing, with no results available to date. The study plans to enroll 40 overweight or obese adults with prediabetes and will investigate the metabolic effects of supplementation with high purity POA capsules (>90% POA as ethyl esters) at a dose of 1,512 mg/day. The prespecified outcomes include insulin sensitivity assessed by the hyperinsulinemic-euglycemic clamp, liver fat quantified by MRI, measures of adiposity, circulating lipids, and inflammatory cytokines.

Overall, human intervention data show that favorable lipid changes arise mainly when POA is consumed within broader food matrices that alter overall dietary fat quality, whereas purified POA supplementation has been largely neutral. Current clinical evidence therefore does not establish a POA-specific lipid-lipoprotein effect, which is why mechanistic studies remain important.

### 3.3 Pre-clinical studies

Across rodent models, POA consistently emerges as a regulator of hepatic lipid handling and lipoprotein metabolism, in a way that broadly aligns with the more modest or neutral lipid effects seen in human supplementation trials. In the original lipokine work, adipose-specific FAS deficiency markedly increased circulating POA and was accompanied by enhanced insulin signaling in skeletal muscle, reduced hepatic steatosis and lower expression of lipogenic genes in liver, indicating that endogenous POA can suppress hepatic *de novo* lipogenesis and limit TG accumulation despite ongoing overnutrition (1).

In diabetic KK-Ay mice, chronic oral administration of POA (300 mg/kg/day for 4 weeks) reduced fasting TG, improved hypertriglyceridemia and diminished hepatic neutral lipid deposition in parallel with down-regulation of SREBP-1, FAS, and SCD1 mRNA, directly implicating POA in the repression of hepatic lipogenic gene expression (36). More recent work in diet-induced obesity extends these observations and begins to map the upstream signaling nodes. In high-fat-fed C57BL/6 mice, POA supplementation increased hepatic AMPK phosphorylation, upregulated glucokinase, reduced SREBP-1 expression and induced FGF-21 production in a PPARα-dependent manner, thereby enhancing hepatic glucose uptake and fatty-acid oxidation while limiting new lipid synthesis (37).

In a complementary model, POA or POA-enriched diets reduced hepatic TG accumulation, ALT activity and histological steatosis, while improving pyruvate tolerance, suggesting a coordinated effect on hepatocellular energy metabolism and protection against NAFLD progression (38, 39). These hepatic effects are mirrored in adipose tissue, where POA increases adipocyte fatty-acid oxidation and TG fatty-acid cycling as well as up-regulating genes involved in lipid mobilization and mitochondrial biogenesis, changes that would be expected to lower the flux of lipids to the liver and circulation over time (39, 40). Pre-clinical atherosclerosis models provide further support for direct vascular benefits. In LDL receptor-deficient mice fed a Western-type diet, replacement of part of the dietary fat with POA attenuated hyperlipidemia, reduced non-HDL cholesterol and aortic lesion area, and increased markers of reverse cholesterol transport and HDL-mediated cholesterol efflux (41).

**Figure 1:**
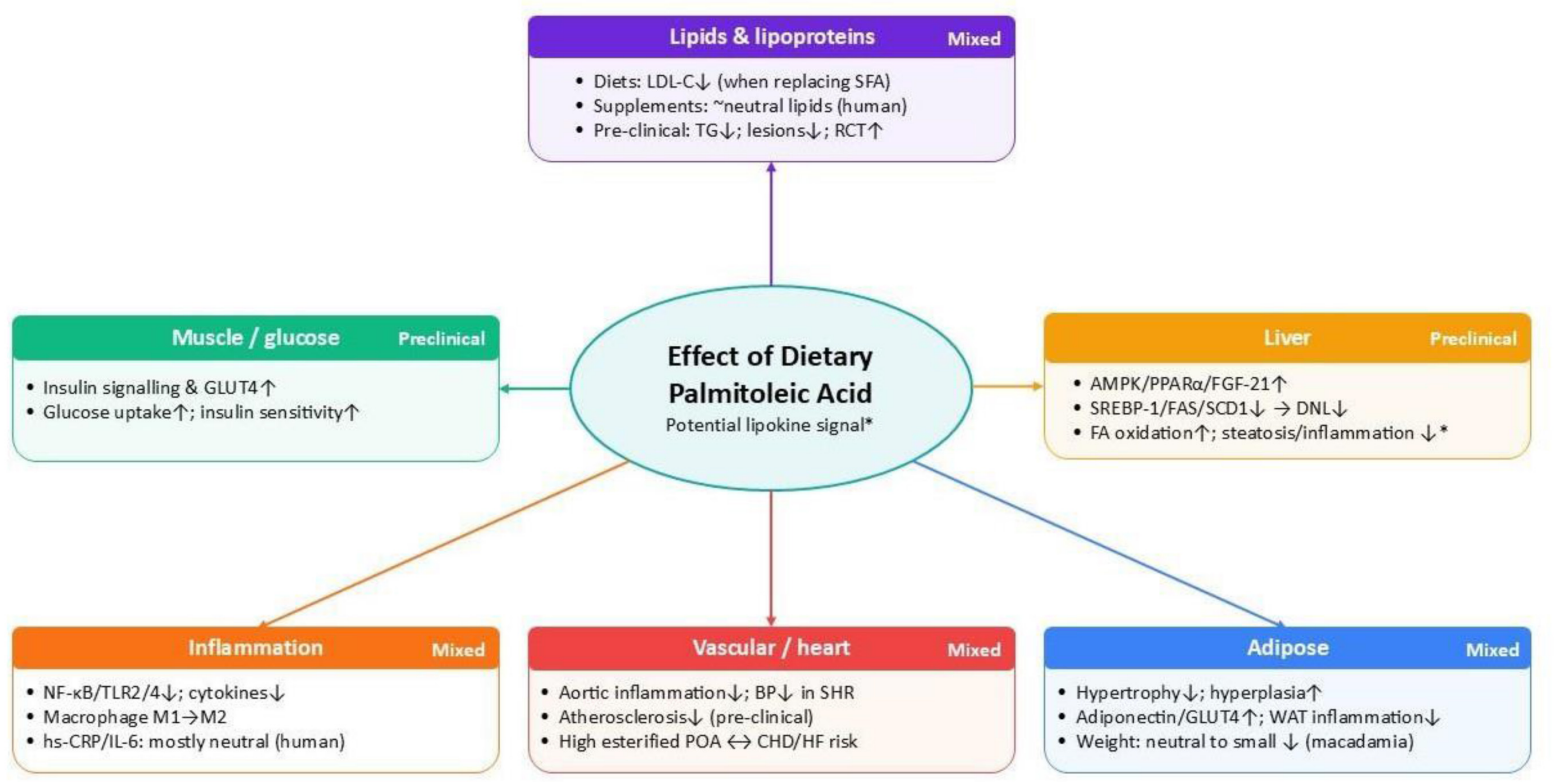
Overview of the effects of dietary palmitoleic acid (POA; 16:1 n-7) across the metabolic domains reviewed. Mixed indicates outcomes from observational, clinical and pre-clinical trials. AMPK, AMP-activated protein kinase; BP, blood pressure; CHD, coronary heart disease; DNL, *de novo* lipogenesis; FA, fatty acid(s); FAS, fatty acid synthase; FGF-21, fibroblast growth factor 21; GLUT4, glucose transporter type 4; HF, heart failure; hs-CRP, high-sensitivity C-reactive protein; IL-6, interleukin-6; LDL-C, low-density lipoprotein cholesterol; M1, classically activated (pro-inflammatory) macrophage phenotype; M2, alternatively activated (anti-inflammatory / pro-resolution) macrophage phenotype; NF-κB, nuclear factor κ-B; PPARα, peroxisome proliferator-activated receptor α; RCT, reverse cholesterol transport; SCD1, stearoyl-CoA desaturase 1; SFA, saturated fatty acid(s); SHR, spontaneously hypertensive rat; SREBP-1, sterol regulatory element-binding protein 1; TG, triglycerides; TLR2/4, Toll-like receptor 2 and Toll-like receptor 4; WAT, white adipose tissue. ^*^Direction and magnitude are context-dependent(dose,background diet,endogenous vs exogenous.

These findings offer a mechanistic rationale for why endogenous POA has sometimes been linked to a more favorable HDL profile and fibrinogen levels, even though purified POA up to 1 g/day has not appreciably altered standard lipid panels in human trials [(34); Figure 1]. At the same time, other mouse studies highlight that the relationship between POA and liver fat is not uniformly beneficial: continuous POA exposure can exacerbate hepatic steatosis while still dampening hepatic inflammatory responses (42), echoing the mixed observational associations between circulating POA and dyslipidemia. Taken together, the pre-clinical data supports a model in which POA primarily modulates hepatic lipid handling and lipoprotein metabolism via AMPK-PPARα/γ-FGF-21 pathways and improved adipose tissue lipid turnover, but the direction of effect likely depends on background diet, dose and whether the signal is driven by endogenous lipogenesis or exogenous supplementation.

## 4 Glucose homeostasis and insulin-related endpoints

### 4.1 Observational and cohort studies

Observational evidence relating POA to glucose homeostasis is strikingly heterogeneous and appears to depend on the lipid fraction and underlying metabolic context. In the Tübingen Lifestyle Intervention Program, circulating free POA in the fasting non-esterified fatty acid (NEFA) fraction of 100 overweight Caucasian adults at high risk of type 2 diabetes correlated positively with insulin sensitivity measured by both Oral Glucose Tolerance Test (OGTT)-derived indices and the hyperinsulinemia-euglycemic clamp, independently of age, sex, and total or visceral adiposity; higher baseline POA also predicted greater improvements in clamp-derived insulin sensitivity after 9 months of lifestyle intervention (9). In contrast, in the Cardiovascular Health Study, higher plasma phospholipid POA was associated with higher Body Mass Index (BMI), waist circumference, and among men, greater insulin resistance, while showing no significant association with incident diabetes, although adjustment for palmitic acid shifted the diabetes hazard ratio below unity (4).

POA level in circulating esterified lipids generally align with worse glycemic profiles. In the Japanese working-population study of 437 adults, higher estimated desaturase activity (reflecting conversion of palmitic to POA) was associated with higher fasting C-peptide concentrations and HOMA2-IR, consistent with greater insulin resistance (6). Among middle-aged and older Chinese adults, erythrocyte POA level was modestly but positively associated with fasting insulin and HOMA-IR after multivariable adjustment, in parallel with its associations with adverse lipid and inflammatory markers (5).

Collectively, these observations suggest that free POA may sometimes reflect a distinct signal associated with higher insulin sensitivity, whereas POA in esterified pools more often clusters with insulin resistance and metabolic syndrome features [(4–6, 9); Figure 1]. This compartment-specific pattern underscores why intervention studies must distinguish endogenous from exogenous POA and specify the lipid pool in which POA is quantified.

### 4.2 Dietary interventions

Glucose homeostasis has been assessed formally in only a subset of trials. In the seabuckthorn oil dose-escalation study at Tufts University, fasting serum glucose and insulin were prespecified secondary outcomes, and no significant changes were detected across any dose of *cis*- or *trans*-POA in metabolically healthy overweight adults [(33); Table 1]. Bridges et al. (34) measured fasting glucose, insulin and HbA1c as secondary metabolic endpoints and found that 500 or 1,000 mg/day POA for 12 weeks did not change these markers relative to placebo in adults with elevated hs-CRP (Table 1). Vashishtha et al. (32) reported fasting glucose as part of baseline characterization and did not describe clinically important changes after 30 days of seabuckthorn seed oil in hypertensive or normotensive cohorts, suggesting no major short-term effect on fasting glycemia in this population (Table 1). No other macadamia or macadamia-oil trials reported detailed glucose or insulin endpoints, so evidence for any direct effect of exogenous POA on insulin sensitivity or glycemic control in humans remains very limited and, to date, largely null [(18, 27–29, 31); Table 1].

An ongoing randomized trial described by Cetin et al. (35) is specifically designed to address this gap: 40 overweight and obese adults with prediabetes in Boston, USA, will receive either 1,512 mg/day of highly purified POA (>90%, < 0.3% palmitic acid) or placebo for 8 weeks, with insulin sensitivity assessed by hyper insulinemic-euglycemic clamp as the primary endpoint. Secondary outcomes include hepatic steatosis, whole-body fat mass, serum lipids and inflammatory markers, but results are not yet available (35).

### 4.3 Pre-clinical and cell culture studies

The strong insulin-sensitizing signal observed for free POA in human observational cohorts is well supported by animal and cell-based mechanistic data. In genetically diabetic KK-Ay mice, chronic oral POA lowered fasting glucose and insulin, improved insulin tolerance, and reduced HOMA-IR, indicating a substantive improvement in whole-body insulin sensitivity (36). These metabolic changes were accompanied by less hepatic lipid accumulation and reduced expression of hepatic SREBP-1, FAS and SCD1, linking improved glycaemia to suppression of both hepatic *de novo* lipogenesis and ectopic fat deposition (36).

In high-fat-fed mice, POA treatment enhanced systemic glucose clearance, increased insulin-stimulated suppression of glycaemia and reduced hepatic gluconeogenesis; at the molecular level, POA increased hepatic AMPK phosphorylation, upregulated glucokinase and FGF-21, and required intact PPARα signaling to exert these effects (37, 43). These pre-clinical findings fit well with the idea emerging from human cohorts that higher free POA (NEFA) may mark a physiological lipokine response that promotes hepatic glucose disposal and limits glucose output, even when fasting plasma glucose remains within the “normal” range.

Skeletal muscle and adipose cell culture studies provide more granular mechanistic support. In rat L6 myotubes, POA prevented palmitate-induced defects in insulin signaling, preserved insulin-stimulated GLUT4 translocation and maintained glucose uptake, whereas palmitic acid alone markedly impaired these endpoints (44).

In 3T3-L1 adipocytes and in adipose tissue from POA-treated mice, POA increased GLUT4 content and glucose uptake via AMPK activation and enhanced adiponectin expression, thereby improving adipocyte insulin responsiveness and potentially augmenting systemic insulin sensitivity (45, 46). Monounsaturated fatty acids, including POA, also protect pancreatic beta-cells from palmitate- and high-glucose-induced apoptosis and dysfunction, preserving insulin secretion in human islets, which offers a further mechanism by which POA could support glucose homeostasis in insulin-resistant states (47).

Collectively, these data offer a coherent mechanistic framework (Figure 1), centered on AMPK activation, improved GLUT4-mediated glucose transport, suppression of hepatic lipogenesis, and preservation of beta-cell integrity. These mechanisms are consistent with the insulin-sensitizing associations of NEFA-POA in observational studies, but they remain to be confirmed in adequately powered randomized trials with direct glycemic endpoints.

## 5 Inflammatory markers and oxidative stress

### 5.1 Observational and cohort studies

Only a subset of observational studies directly assessed inflammatory biomarkers alongside POA, but available data indicate that higher POA often co-varies with low-grade inflammation. In the large Chinese cohort with erythrocyte fatty acids, POA was independently and positively associated with hs-CRP, plasminogen activator inhibitor-1, retinol-binding protein-4 and elevated blood pressure after adjustment for adiposity, lifestyle and diet, while being inversely related to adiponectin (5). In the Tübingen cohort, by contrast, free POA was unrelated to hs-CRP despite its strong positive association with insulin sensitivity, suggesting that under some conditions, POA may be dissociated from systemic inflammatory burden (9).

Vascular inflammatory pathways have been probed more mechanistically in the combined human-animal study by Tang et al. (48). In a case-control analysis nested within a school-based cohort of 5,267 Chinese children and adolescents, hypertensive cases had significantly lower erythrocyte phospholipid POA than normotensive controls, and participants in the top POA quartile had roughly 50% lower odds of primary hypertension than those in the bottom quartile after multivariable adjustment (48). Taken together, epidemiological studies suggest that higher POA in some lipid fractions may correlate with pro-inflammatory states (5), whereas experimental data support direct anti-inflammatory actions of exogenous POA on the vascular wall (48); the balance between these patterns likely depends on whether POA reflects overactive SCD1-driven lipogenesis vs. a compensatory lipokine signal.

Given that higher POA in erythrocyte and plasma esterified fractions frequently co-varies with hs-CRP, pro-thrombotic adipokines and blood pressure, it is relevant to assess whether increasing POA intake can attenuate systemic inflammation in randomized clinical trials, or whether circulating POA mainly reports on an underlying pro-inflammatory, lipogenesis-driven condition.

### 5.2 Dietary interventions

Bridges trial found no effect of 500 or 1,000 mg/day marine-derived POA on hs-CRP, IL-6, or TNF-α relative to olive-oil placebo in adults with hs-CRP >=2 mg/L [(34); Table 1]. Huang et al. (33) likewise detected no meaningful changes in routine inflammatory or clinical chemistry measures in their dose-escalation trial of *cis*- or *trans*-POA-enriched seabuckthorn oil, although the study was not powered for these outcomes (Table 1). Similarly, a randomized crossover trial of a mixed omega-7 supplement containing 688 mg/day palmitoleate reported no reductions in hs-CRP, TNF-α, or IL-6 over 3 weeks (49).

In the high-altitude Indian cohort, seabuckthorn seed oil supplementation reduced oxidized LDL and increased total antioxidant capacity in both hypertensive and normotensive groups, indicating an improvement in oxidative stress markers [(32); Table 1]. Given the substantial vitamin E and carotene content of the oil, these changes are likely driven by innate antioxidants as well as unsaturated fatty acids, including POA, contained in the oil (32).

Taken together, current human supplementation trials do not show consistent reductions in systemic inflammatory biomarkers, even though some food-based studies report favorable changes in oxidative-stress markers. This gap between mechanistic plausibility and clinical biomarker responses makes tissue-level pre-clinical data relevant, while also arguing for cautious interpretation of efficacy claims.

### 5.3 Pre-clinical and cell culture trials

Although large POA supplementation trials in humans have not yet demonstrated clear reductions in circulating CRP or cytokines, pre-clinical work shows robust anti-inflammatory actions of POA in metabolically relevant tissues and cell types. In high-fat-fed mice, POA supplementation lowered circulating pro-inflammatory cytokines, improved insulin sensitivity and reduced liver and adipose tissue inflammatory gene expression, despite no major changes in body weight (43). In a follow-up study focused on NAFLD, POA treatment reduced hepatic MCP-1 and TNF-α levels, decreased ALT and histological steatosis, and shifted liver macrophages from a pro-inflammatory M1-like phenotype toward an anti-inflammatory, CD206-positive M2a profile, thereby dampening local inflammatory tone while simultaneously improving glucose tolerance (38).

At the cellular level, POA directly modulates macrophage polarization and inflammatory signaling. In bone-marrow-derived macrophages from high-fat-fed mice, palmitoleate activated AMPK and reversed the high-fat, diet-induced shift toward a pro-inflammatory M1 phenotype, reducing expression and secretion of TNF-α, IL-6 and other M1 markers while increasing markers of oxidative metabolism associated with M2 polarization (50).

In J774 macrophages and rat peritoneal macrophages, POA attenuated palmitate- and LPS-induced inflammation, down-regulated TLR2 and TLR4 expression and promoted an alternative activation program, effects that were at least partly independent of TNF-α and consistent with a direct counter-regulatory role on saturated-fat-driven TLR-NF-κB signaling (37, 51). POA also reduces NF-κB activation and pro-inflammatory gene expression in LPS-stimulated macrophages *via* AMPK-dependent mechanisms, while increasing the anti-inflammatory cytokine IL-10, further underscoring its capacity to re-program innate immune responses toward a resolving phenotype (37).

Consistent with these findings, adipose tissue and stromal vascular fraction from high-fat- fed mice treated with POA show lower expression of Mcp1, Tnfα, Il6, Cxcl10, and Nos2 compared with untreated obese controls, indicating that POA can dampen the low-grade inflammation characteristic of dysfunctional visceral fat (46).

These anti-inflammatory effects extend to adipose-derived stromal cells and may be an important part of how POA reshapes the adipose micro-environment. POA-treated obese mice exhibit reduced adipocyte hypertrophy, increased adipocyte hyperplasia and increased expression of PPARγ2, Cebpα, Fabp4, Plin1, Glut4, and AdipoQ in adipose tissue, a pattern consistent with the emergence of smaller, more insulin-sensitive adipocytes that are less prone to hypoxia, oxidative stress and chemokine production (40, 46). Macadamia-oil supplementation, a natural source of POA along with other MUFA, similarly reduces adipocyte hypertrophy and adipose inflammatory markers in diet-induced obese mice, suggesting that POA-rich food matrices may foster a less inflammatory adipose phenotype *in vivo* (52).

Altogether, these mechanistic data provide a plausible biological basis for modest, tissue-level anti-inflammatory actions of POA that may not be fully captured by systemic biomarkers such as hs-CRP in relatively healthy human trial populations (Figure 1).

## 6 Blood pressure and vascular endpoints

### 6.1 Observational and cohort studies

The observational literature on POA and blood pressure is limited but points to potentially divergent associations across age groups and vascular outcomes. In Chinese school-aged children and adolescents, erythrocyte phospholipid POA was inversely related to the prevalence of primary hypertension: hypertensive cases had significantly lower POA than normotensive controls, and those in the highest POA quartile had a multivariable-adjusted odds ratio of 0.47 (95% CI 0.25–0.89) for hypertension compared with the lowest quartile (48). In the same study, POA supplementation in spontaneously hypertensive rats lowered systolic blood pressure and improved aortic remodeling, suggesting a plausible causal pathway via suppression of NF-κB-mediated vascular inflammation (48). In adults, POA appears more consistently linked to adverse vascular outcomes. In the Physicians' Health Study, higher plasma phospholipid POA was prospectively associated with an increased risk of incident heart failure over more than 22 years, independent of traditional cardiovascular risk factors and other fatty acids (7). A nested case-control analysis in the same cohort also reported that higher red-blood-cell POA was associated with elevated risk of coronary heart disease (7).

In cross-sectional work, plasma POA content in French men correlated positively with systolic blood pressure and TG and negatively with HDL-cholesterol, clustering with features of the metabolic syndrome (3), while erythrocyte POA in Chinese adults showed modest positive correlations with both systolic and diastolic blood pressure (5). Overall, these findings suggest that circulating POA may be inversely associated with hypertension in pediatric populations but positively associated with long-term heart failure and coronary heart disease risk in older adults, again highlighting the importance of the underlying metabolic background and the lipid compartment in which POA is measured.

The mixed observational picture, in which higher POA is inversely associated with primary hypertension in children but clusters with higher blood pressure and increased long-term risk of CHD and heart failure in adults, provides an important framework for evaluating whether POA-rich dietary interventions can translate into blood-pressure lowering or improved vascular risk profiles in clinical settings.

### 6.2 Dietary interventions

In the Ladakh seabuckthorn seed oil trial, hypertensive, hypercholesterolemic men who consumed seabuckthorn seed oil (0.75 mL/day) for 30 days experienced statistically significant reductions in both systolic and diastolic blood pressure compared with baseline and with placebo sunflower oil [(32); Table 1]. No meaningful blood pressure changes occurred in normotensive men supplemented with seabuckthorn seed oil, indicating effects primarily in hypertensive individuals (32). Curb et al. (18) and Hiraoka-Yamamoto et al. (29) measured blood pressure in their macadamia interventions and reported values within normal limits with no significant between-diet differences over 3–4 weeks, suggesting no short-term pressor or depressor effect of macadamia-rich diets in normotensive populations (Table 1). Bridges et al. (34) assessed blood pressure at clinic visits but did not report any treatment-related changes over the 12-week POA supplementation period (Table 1).

Because macadamia-based diets and purified POA capsules have generally had little impact on blood pressure in normotensive adults, and seabuckthorn seed oil has shown blood pressure normalization only in hypertensive subjects receiving a complex mix of fatty acids and antioxidants, vascular and cardiac models are particularly informative to determine whether POA itself exerts direct effects on the arterial wall and myocardium beyond its influence on classical risk factors.

### 6.3 Pre-clinical studies

Direct pre-clinical data on POA and blood pressure are limited, but several vascular and cardiac models support a role for POA in modulating vascular inflammation, atherogenesis and myocardial stress responses. In LDL receptor-deficient mice, dietary POA not only improved the plasma lipid profile but also reduced aortic atherosclerotic lesion area and macrophage infiltration, concomitant with down-regulation of vascular adhesion molecules and inflammatory mediators, suggesting that POA can attenuate the vascular inflammatory conditions that drives plaque progression (41).

In spontaneously hypertensive rat models referenced alongside the human observational work, chronic POA administration lowered systolic blood pressure and improved aortic remodeling, effects linked to suppression of NF-κB-mediated vascular inflammation and oxidative stress (48). These findings are broadly consistent with the inverse association between erythrocyte phospholipid POA and hypertension observed in children but stand in contrast to the generally null blood-pressure findings in short-term human supplementation trials. In the heart, POA appears to act as a lipid signal that can influence both adaptive and stress-induced remodeling. In a catecholamine-induced cardiac injury model, POA treatment exerted cardioprotective effects, attenuating myocardial damage via PPAR-mediated pathways and improving indices of cardiac function (51).

In a separate model of exercise-induced physiological hypertrophy, adipose tissue lipolysis augmented circulating POA, which contributed to adaptive cardiac growth and improved cardiac performance, highlighting POA as a mediator linking adipose metabolism, vascular function and myocardial remodeling (53).

These mechanistic data support the concept that endogenous POA may modulate vascular inflammation, arterial structure and cardiac stress responses, but they also underscore that such effects are context-dependent and tightly intertwined with overall adipose and lipid metabolism, features that may help explain why circulating POA in adults sometimes correlates with higher risk of heart failure and CHD in epidemiological studies despite potentially beneficial actions in specific experimental settings (Figure 1).

## 7 Body weight and adiposity

### 7.1 Observational and cohort studies

Across age groups and tissues, higher circulating POA is generally associated with greater adiposity, particularly central obesity. In a Japanese case-control study of 59 obese and 53 non-obese children, plasma POA and the SCD1 activity index (POA to palmitic acid ratio, 16:1 to 16:0) were markedly higher in obese children; in multivariable models, percentage body fat and waist-to-height and waist-to-hip ratios were significant determinants of plasma POA, and SCD1 activity correlated positively with leptin but was ultimately best explained by central adiposity (54). In French adult men, plasma POA content was strongly associated with waist circumference, and remained an independent predictor of increased abdominal adiposity even after accounting for TG levels and other metabolic risk factors (3). Adipose-tissue POA has also been examined directly. In a population-based Costa Rican sample of 1,926 adults, higher POA concentrations and higher SCD1 desaturation indices (16:1/16:0 and 18:1/18:0) in subcutaneous adipose tissue were strongly associated with the prevalence of obesity; compared with the lowest quintile, participants in the top quintile of adipose POA had roughly a 4-fold higher prevalence of obesity, with the association being most pronounced among those with higher carbohydrate intake (55). In the Cardiovascular Health Study, higher circulating phospholipid POA was associated with higher BMI and larger waist circumference in both sexes (4), whereas in the Tübingen cohort, free POA was not significantly correlated with measures of total or visceral fat mass despite its strong association with insulin sensitivity (9).

Collectively, these observational data support the view that higher POA in plasma phospholipids, cholesterol esters, or adipose tissue is a robust marker of increased adiposity and SCD1-driven lipogenesis, while free POA in the NEFA fraction may sometimes capture a more distinct signal not tightly coupled to fat mass. These associations also make it important to determine whether POA-rich foods or supplements can be consumed without promoting weight gain in intervention studies.

### 7.2 Dietary interventions

Most trials were not designed as weight-loss interventions and attempted to keep energy intake stable, but they nevertheless provide some information on body weight effects. Garg et al. (28) observed a small but statistically significant decrease in body weight and BMI over 4 weeks of macadamia nut supplementation in hypercholesterolemic men, despite the nuts being energy-dense, suggesting some degree of compensation in energy intake or increased satiety (Table 1). In the controlled feeding trial by Griel et al. (27), energy intake was tightly regulated to maintain body weight, and body weight remained stable across both the macadamia and average American diets, indicating that macadamia inclusion *per se* did not promote weight gain when substitutions were isocaloric (Table 1). Hiraoka-Yamamoto et al. (29) found that 3 weeks of macadamia-nut bread significantly reduced body weight and BMI in young Japanese women, whereas coconut and butter breads did not, even though macadamia bread was not lower in energy than the comparison breads (Table 1). This suggests that high-MUFA, POA-rich macadamia fat may support modest weight reduction or improved body composition in free-living conditions, although mechanisms were not explored (29). In contrast, seabuckthorn and purified POA supplement trials generally did not report significant changes in body weight, consistent with their design as metabolic biomarker rather than weight-loss studies [(32–34); Table 1].

The small weight reductions seen in some macadamia studies and the largely neutral weight trajectories in seabuckthorn and purified POA trials do not provide convincing evidence that POA promotes weight loss in humans. Animal studies instead suggest effects on adipose remodeling and satiety signaling that remain hypotheses to be tested clinically.

### 7.3 Pre-clinical studies

Pre-clinical models provide a coherent picture in which POA does not necessarily reduce total fat mass dramatically but remodels adipose tissue toward a “healthier” phenotype and modestly attenuates weight gain under obesogenic conditions. In high-fat-fed mice, oral POA reduced body-weight gain by about one-third over a 4-week treatment period and completely prevented epididymal adipocyte hypertrophy, despite leaving total epididymal fat mass largely unchanged, findings that point to increased adipocyte hyperplasia and improved storage capacity rather than total fat loss (46). POA treatment increased proliferation and differentiation of 3T3-L1 pre-adipocytes, increased expression of master adipogenic regulators PPARγ2 and Cebpα, and up-regulated key terminal differentiation markers (Fabp4, Plin1, Glut4, and AdipoQ), consistent with the generation of smaller, insulin-sensitive adipocytes capable of buffering lipid overflow and limiting ectopic fat deposition (45, 46). In a related model, POA increased adipocyte fatty-acid oxidation and lipogenesis while blunting the high-fat-induced overexpression of genes involved in lipolysis and inflammation, suggesting that POA facilitates metabolic flexibility in adipose tissue and reduces the need for “emergency” lipolytic responses that can drive lipotoxicity (39).

These adaptations are conceptually compatible with clinical and observational data in which higher POA is strongly associated with adiposity and SCD1 activity yet may denote a metabolically more “plastic” adipose depot in some individuals. POA may also influence body weight indirectly through appetite regulation and energy intake. In male rats, acute oral administration of POA reduced subsequent food intake and body-weight gain and increased circulating satiety hormones, including cholecystokinin and peptide YY; these anorectic effects were attenuated by pharmacological CCK-1 receptor blockade, implicating gut-brain satiety pathways in the response to POA [(56); Figure 1].

## 8 Conclusion

Taken together, the evidence supports a context-dependent interpretation of POA. Higher POA in esterified circulating pools often tracks with SCD1-driven lipogenesis and adverse cardiometabolic phenotypes, whereas free POA in some settings has been associated with greater insulin sensitivity. Human observational data therefore do not justify assuming that higher circulating POA is intrinsically beneficial. The clearest human intervention signal is that macadamia-based dietary patterns can improve LDL-cholesterol when they replace saturated fat, but these studies test a mixed monounsaturated-fat food matrix and do not isolate POA-specific effects. By contrast, purified POA supplementation has reliably increased circulating POA status yet has generally shown neutral effects on standard fasting lipids, glycemic markers, and systemic inflammatory biomarkers. Pre-clinical studies still provide a rationale for further work because they support effects on hepatic lipid handling, insulin signaling, adipose remodeling, and inflammatory pathways. Future randomized trials should focus on clearly defined at-risk populations, longer intervention periods, careful control of background diet, and outcomes capable of distinguishing endogenous from exogenous POA and detecting effects beyond routine fasting biomarkers. Until such data are available, POA should be viewed as biologically interesting but clinically unproven as a standalone metabolic intervention.

## Glossary

**AAD** average American diet

**ALT** alanine aminotransferase

**AMPK** AMP-activated protein kinase

**BMI** body mass index

**BP** blood pressure

**CCK** cholecystokinin

**CD206** cluster of differentiation 206

**CHD** coronary heart disease

**CI** confidence interval

**CRP** C-reactive protein

**FAS** fatty acid synthase

**FFA** free fatty acids

**FGF-21** fibroblast growth factor 21

**GLUT4** glucose transporter type 4

**HDL** high-density lipoprotein

**HDL-C** high-density lipoprotein cholesterol

**HOMA-IR** homeostatic model assessment of insulin resistance

**HOMA2-IR** homeostatic model assessment 2 of insulin resistance

**ICAM-1** intercellular adhesion molecule 1

**IL-10** interleukin 10

**IL-6** interleukin 6

**KK-Ay** mouse model of obesity/type 2 diabetes

**LDL** low-density lipoprotein

**LDL-C** low-density lipoprotein cholesterol

**LPS** lipopolysaccharide

**MAC** macadamia-nut-rich diet

**MCP-1** monocyte chemoattractant protein 1

**MCT** medium-chain triglycerides

**MRI** magnetic resonance imaging

**MUFA** monounsaturated fatty acid(s)

**NAFLD** non-alcoholic fatty liver disease

**NASH** non-alcoholic steatohepatitis

**NEFA** non-esterified fatty acids

**NF-κ**B nuclear factor κ-light-chain-enhancer of activated B cells

**OGTT** oral glucose tolerance test

**PAI-1** plasminogen activator inhibitor 1

**PLFA** plasma lipid fatty acids

**POA** palmitoleic acid

**PPAR** peroxisome proliferator-activated receptor

**PPARα** peroxisome proliferator-activated receptor α

**PPARγ** peroxisome proliferator-activated receptor γ

**RBP-4** retinol-binding protein 4

**SCD1** stearoyl-CoA desaturase 1

**SREBP-1c** sterol regulatory element-binding protein 1c

**TG** triglycerides

**TLR** Toll-like receptor

**TLR2** Toll-like receptor 2

**TLR4** Toll-like receptor 4

**TNF-α** tumor necrosis factor α
